# Identification of an epigenetic biomarker panel with high sensitivity and specificity for colorectal cancer and adenomas

**DOI:** 10.1186/1476-4598-10-85

**Published:** 2011-07-21

**Authors:** Guro E Lind, Stine A Danielsen, Terje Ahlquist, Marianne A Merok, Kim Andresen, Rolf I Skotheim, Merete Hektoen, Torleiv O Rognum, Gunn I Meling, Geir Hoff, Michael Bretthauer, Espen Thiis-Evensen, Arild Nesbakken, Ragnhild A Lothe

**Affiliations:** 1 Department of Cancer Prevention, Institute for Cancer Research, Oslo University Hospital - Radiumhospitalet, Oslo, Norway; 2 Centre for Cancer Biomedicine, Faculty of Medicine, University of Oslo, Oslo, Norway; 3 Surgical Department, Oslo University Hospital - Aker Hospital, Oslo, Norway; 4 Institute of Forensic Medicine, University of Oslo, Norway; 5 Faculty of Medicine, University of Oslo, Oslo, Norway; 6 Department of Medicine Division of Gastroenterology, Telemark Hospital, Skien, Norway; 7 The Cancer Registry of Norway, Oslo, Norway; 8 Department of Gastroenterology, Oslo University Hospital, Oslo, Norway; 9 Department for organ transplantation, gastroenterology and nephrology, Oslo University Hospital - Rikshospitalet, Oslo, Norway; 10 Research Center, Akershus University Hospital, Lørenskog, Norway

**Keywords:** Biomarker, CNRIP1, colorectal neoplasia, early detection, FBN1, INA, MAL, methylation, SNCA, SPG20

## Abstract

**Background:**

The presence of cancer-specific DNA methylation patterns in epithelial colorectal cells in human feces provides the prospect of a simple, non-invasive screening test for colorectal cancer and its precursor, the adenoma. This study investigates a panel of epigenetic markers for the detection of colorectal cancer and adenomas.

**Methods:**

Candidate biomarkers were subjected to quantitative methylation analysis in test sets of tissue samples from colorectal cancers, adenomas, and normal colonic mucosa. All findings were verified in independent clinical validation series. A total of 523 human samples were included in the study. Receiver operating characteristic (ROC) curve analysis was used to evaluate the performance of the biomarker panel.

**Results:**

Promoter hypermethylation of the genes *CNRIP1, FBN1, INA, MAL, SNCA*, and *SPG20 *was frequent in both colorectal cancers (65-94%) and adenomas (35-91%), whereas normal mucosa samples were rarely (0-5%) methylated. The combined sensitivity of at least two positives among the six markers was 94% for colorectal cancers and 93% for adenoma samples, with a specificity of 98%. The resulting areas under the ROC curve were 0.984 for cancers and 0.968 for adenomas versus normal mucosa.

**Conclusions:**

The novel epigenetic marker panel shows very high sensitivity and specificity for both colorectal cancers and adenomas. Our findings suggest this biomarker panel to be highly suitable for early tumor detection.

## Background

Colorectal cancer is the third most common cancer type in the US and is a major contributor to cancer-death [1]. Most cases of colorectal cancer develop from benign precursors (adenomas) during a long time interval. This provides a good opportunity for detection of colorectal cancer at an early curable stage and to screen for potentially pre-malignant adenomas [2]. Both flexible sigmoidoscopy and the Fecal Occult Blood Test (FOBT) have been tested in randomized trials and shown to reduce mortality from colorectal cancer [3]. By sigmoidoscopy adenomas may be detected and removed and thus the incidence of cancer will be reduced [4], however, this screening is invasive and cumbersome for the patient. FOBT on the other hand is non-invasive and currently the most commonly used screening test for colorectal cancer in Europe. Although the sensitivity and specificity measurements of FOBT have been substantially improved in recent years [5], they are still not optimal. FOBT is also hampered by the low sensitivity for adenomas. Therefore, during recent years, much effort has been put in the development of fecal DNA markers. A successful biomarker panel that is able to discriminate between healthy individuals and carriers of early colorectal cancer or precursor lesions has the potential of reducing both incidence and mortality of the disease. Until today, however, no feces DNA test has achieved a satisfactory performance level compared to the screening tests mentioned above.

Aberrant DNA promoter methylation has previously been shown to be an early event in the development of colorectal cancer [6-10]. Several reports of DNA methylation biomarkers tested in fecal [8,11-15] and blood samples [16-19] suggest the suitability of epigenetic biomarkers in early diagnostics of the disease. However, only markers which provide a high methylation frequency in samples from colorectal cancer patients and at the same time lack hypermethylation in normal mucosa are suitable for a screening test.

The present study reports on the performance (the sensitivity and specificity) of a novel epigenetic biomarker panel.

## Methods

### Selection of epigenetic markers analyzed in the present study

**Figure 1:**
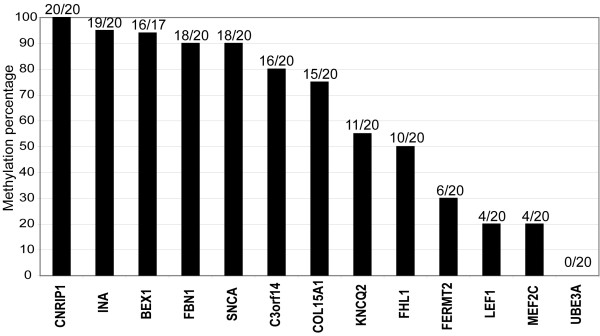
**DNA promoter hypermethylation status of 13 candidate genes in colon cancer cell lines**. Colon cancer cell lines were used as *in vitro *models to explore the DNA promoter methylation frequency of 13 candidate biomarkers. Only candidates with methylation frequencies equal to or higher than 80% (*CNRIP1, INA, BEX1, FBN1, SNCA*, and *C3orf14*) were subjected to methylation analysis in tissue samples.

From an epigenomic screen of colon cancer *in vitro *models we have previously identified a number of genes responding to 5-aza-2'deoxycytidine treatment [20]. In the present study, thirteen of these candidates were analyzed in 20 colon cancer cell lines in order to identify the most suitable DNA methylation markers for colorectal cancer (Table 1, Figure 1). From this analysis we selected *CNRIP1, FBN1, INA*, and *SNCA *for detailed studies in clinical sample series using quantitative MSP (qMSP) assays. Additionally, a qMSP assay was designed and applied for analysis of the *MAL *gene promoter, previously reported by us as a biomarker for early detection of colorectal tumors by qualitative MSP [21,22]. Finally, the *SPG20 *biomarker recently reported with a sensitivity of 89% and 78% in colorectal cancer and adenomas, respectively and a specificity of 99% [9] was included for evaluation of a combined biomarker panel performance.

**Table 1:** Names, chromosomal location, and sequence accession number for genes analyzed in the present study

Gene Symbol^a^	Gene Name^a^	Chromosomal Location^a^	Accession Number^b^
*BEX1*	brain expressed, X-linked 1	Xq21-q23	NM_018476
*C3orf14*	chromosome 3 open reading frame 14	3	NM_020685
*CNRIP1*	cannabinoid receptor interacting protein 1	2p13	NM_015463
*COL15A1*	collagen, type XV, alpha 1	9q21-q22	NM_001855
*FBN1*	fibrillin 1	15q21.1	NM_000138
*FERMT2*	fermitin family homolog 2 (Drosophila)	14q22.1	NM_006832
*FHL1*	four and a half LIM domains 1	Xq26.3	NM_001449
*INA*	internexin neuronal intermediate filament protein, alpha	10q24	NM_032727
*KCNQ2*	potassium voltage-gated channel, KQT-like subfamily, member2	20q13.33	NM_172106
*LEF1*	lymphoid enhancer-binding factor 1	4q23-q25	NM_016269
*MEF2C*	myocyte enhancer factor 2C	5q14	NM_002397
*SNCA*	synuclein, alpha (non A4 component of amyloid precursor)	4q21.3-q22	NM_000345
*UBE3A*	ubiquitin protein ligase E3A	15q11-q13	NM_130839

^a^Gene Symbols, full Gene Name, and Chromosome location are in accordance with the approved guidelines from the HUGO Gene Nomenclature Committee at the European Bioinformatics Institute, http://www.genenames.org

^b^Sequence Accession Numbers are from the USCS Genome Browser http://genome.ucsc.edu/ and represent sequences used for primer design.

### Cancer Cell Lines

Nine of the 20 colon cancer cell lines were microsatellite unstable, MSI (Co115, HCT15, HCT116, LoVo, LS174T, RKO, SW48, TC7, and TC71), and 11 were microsatellite stable, MSS (ALA, Colo320, EB, FRI, HT29, IS1, IS2, IS3, LS1034, SW480, and V9P) [23]. Culturing conditions included DMEF-12 medium (GIBCO, Invitrogen Carlsbad, CA) with 15% fetal bovine serum (GIBCO, 2 mM L-glutamine (GIBCO), 100 U/ml penicillin G, and 100 μg/ml streptomycin (GIBCO).

Three MSI cell lines (HCT15, RKO, and SW48) and three MSS cell lines (HT29, LS1034, and SW480) were treated with a) 1 μM of 5-aza-2'deoxycytidine (Sigma-Aldrich, St. Louis, MO, USA) for 72 h, b) 0.5 μM of trichostatin A (Sigma-Aldrich) for 12 h, and c) a combination of both drugs (1 μM 5-aza-2'deoxycytidine for 72 h, 0.5 μM trichostatin A added the last 12 h). The medium was exchanged daily.

Twenty-nine additional cell lines from various cancer tissues other than colon cancer were included (described in Additional file 1). All cell lines were harvested before reaching confluence. DNA was extracted from the cancer cell lines by using a standard phenol-chloroform procedure [24], and total RNA was isolated using Trizol (Invitrogen, Carlsbad, CA).

### Colorectal cancer and adenoma samples

The colorectal cancer test set comprised DNA from 79 fresh-frozen cases from 78 patients derived from a prospective series from seven hospitals in South-East Norway during 1987-1989 [25]. Twenty-eight tumors were MSI, and 51 were MSS [26,27]. The median patient age at diagnosis was 71 years (range 33-92 years). One patient fulfilled the criteria for hereditary non-polyposis colorectal cancer (HNPCC) [26]. The colorectal cancer validation set consisted of an independent cohort of 105 cases obtained from a prospective series of fresh-frozen colorectal cancer samples at the Department of Surgery, at Oslo University Hospital - Aker Hospital in the period of 2005-2007. Twenty-two out of 105 (21%) cancers were MSI-high, whereas the remaining 83 (79%) samples were of the MSS or MSI-low phenotype. The median patient age at surgery was 71 years (range 29-93 years).

A series of 61 adenomas was obtained from 50 individuals attending a population-based colonoscopy screening study (Telemark, Norway) [28]. The median age at adenoma removal was 67 years (range 62-72 years) and the median adenoma size was 8 mm (range 5-50 mm). Two of the adenomas were MSI whereas the remaining 59 were MSS [20]. The validation set comprised 51 adenomas from 46 individuals attending another screening study [29]. The median age at adenoma removal was 58 years (range 50-64 years) and the median adenoma size was 11 mm (range 4-40 mm).

### Normal colorectal tissue samples

Two cohorts of normal colorectal mucosa samples were analyzed. The test set consisted of 51 samples from 48 deceased colorectal cancer-free individuals collected at the Institute of Forensic Medicine, University of Oslo. The median age was 55 years (range 22-86 years). The validation set consisted of rectal mucosa biopsies from 59 individuals attending the population-based colonoscopy screening study mentioned above, harboring neither colorectal adenomas nor cancers [28]. The median age was 67 years (range 63-72 years). Also, 105 normal colorectal mucosa samples taken from the resection margin of the colorectal cancer validation series were included in the present study. Median age for these was 71 years.

### Bisulfite Treatment

DNA (1.3 μg) from each tissue sample was bisulfite treated using the EpiTect bisulfite kit (Qiagen) according to the manufacturer's protocol. The desulfonation and washing steps were performed using a QiaCube (Qiagen).

### Qualitative methylation-specific polymerase chain reaction (MSP)

Qualitative methylation-specific polymerase chain reaction (MSP) primers were designed using Methyl Primer Express v1.0 (Applied Biosystems, Foster City, CA, USA) according to the following criteria: primers amplified a region within 300 bases of the annotated transcription start site (UCSC Genome Browser [30]), the maximum fragment length was 200 bp, each primer contained a minimum of two CpG sites, all primer pairs contained a minimum of five Cs in non-CpG sites, and all forward primers had a 3'-proximal CpG site. Primers were purchased from MedProbe (MedProbe, Oslo, Norway) and their sequences are listed in Additional file 1, Table S1, along with the product fragment lengths, primer locations, MgCl_2 _reaction concentration, and PCR annealing temperature. The MSP templates were amplified using the HotStarTaq DNA polymerase (Qiagen). Human placental DNA (Sigma-Aldrich) treated *in vitro *with *Sss1 *methyltransferase (New England Biolabs, Ipswich, MA, USA) was used as a positive control for the methylated MSP reaction, whereas DNA from normal lymphocytes was used as a positive control for the unmethylated reaction. In both reactions we used water as a negative control. PCR products were separated by electrophoresis using 2% agarose and visualized by UV irradiation using a Gene Genius (Syngene, Frederick, MD, USA). All results were confirmed by a second independent round of MSP and scored independently by two authors (SAD and GEL). A third independent MSP was done for samples with diverging results or discrepant scoring from the two initial runs. For each gene, the MSP controls were sequenced in order to verify the identity of the amplified product.

### Quantitative methylation-specific polymerase chain reaction (qMSP)

*CNRIP1, FBN1, INA, MAL*, and *SNCA *promoter methylation were analyzed by qMSP in the test and validation sets as well as in stool samples. *SPG20 *has previously been reported [9]. Primers and probes were designed using Primer express v3.0 (Applied Biosystems) and purchased from MedProbe (MedProbe, Oslo, Norway) and Applied Biosystems, respectively. Sequences are listed in Additional file 1, Table S2. Probes were labelled by 6-FAM and a minor groove binder non-fluorescent quencher. All genes were amplified and normalized for DNA input using the *ALU-C4 *sequence [31]. The qMSP was carried out in triplicates in 384-well plates using a 20 μl reaction volume including 0.9 μM each of forward and reverse primers, 0.2 μM probe, 30 ng bisulfite treated template (tissue samples) or 1 ul bisulfite treated template (stool sample), and 1 × TaqMan Universal PCR master mix NoAmpErase UNG (including AmpliTaq Gold DNA polymerase and passive reference; ROX). Fragments were amplified at 95°C for 10 min, then 45 cycles of 95°C for 15 sec followed by 60°C for 1 min using the 7900HT Sequence Detection System (Applied Biosystems). The median value was used for data analysis. Bisulfite-converted completely methylated DNA (CpGenome Universal Methylated DNA; Millipore Billerica, MA, USA) served as a positive control for the qMSP reaction and 1:5 serial dilutions (32.5 - 0.052 ng) were used to generate a standard curve for quantification. Additionally, all plates contained multiple water blanks, bisulfite modified DNA from normal lymphocytes as well as unmodified DNA as a negative control.

For all samples, amplification after cycle 35 was censored, in accordance with the protocol from the manufacturer (Applied Biosystems). The qMSP results were calculated as percent of methylated reference (PMR) in accordance with a previous report [32]. In brief, the median GENE:ALU ratio of a sample was divided by the median GENE:ALU ratio of the positive control (CpGenome Universally Methylated DNA) and multiplied by 100. To ensure high specificity for tissue samples, the percentile of the highest PMR value across all genes and all normal mucosa samples in the test set was used to set a fixed threshold (at PMR = 7) for scoring positive methylation, regardless of the gene in question. This scoring threshold was used in analyses of all tissue samples, including normal mucosa, adenomas and carcinomas in both the test and validation sets. Three samples had outlier PMR values (*CNRIP1*, 12.41; *FBN1*, 12.00; and *MAL*, 12.22) that were excluded when the fixed threshold was set. The validation sets and the normal mucosa samples matching the CRC validation set were analyzed in a blinded manner.

### Direct bisulfite DNA sequencing

DNA bisulfite primers were designed using Methyl Primer Express v1.0 (Applied Biosystems) according to the following criteria: sequences covered the area amplified by the MSP primers of the respective gene promoter including the transcription start point, maximum fragment length was 450 bp, preferably none and maximum two CpG sites were included in each primer, and when possible, repetitive sequences of more than eight bases were avoided. Primer sequences, product fragment lengths, primer locations, MgCl_2 _reaction concentration, and PCR annealing temperatures are listed in Additional file 1, Table S1. A representative promoter region of *CNRIP1, FBN1, INA*, and *SNCA *was subjected to direct bisulfite sequencing in 20 colon cancer cell lines, as previously described [22]. The approximate amount of methyl cytosine of each CpG site was calculated by comparing the peak height of the cytosine signal with the sum of the cytosine and thymine peak height signals [33]. CpG sites with ratios ranging from 0-0.20 were classified as unmethylated, CpG sites within the range 0.21-0.80 were classified as partially methylated, and CpG sites ranging from 0.81-1.0 were classified as hypermethylated.

### RNA isolation, cDNA preparation, and real-time quantitative gene expression analysis

Total RNA was extracted from cancer cell lines (n = 47), colorectal cancers (CRC test set; n = 17), and normal colorectal tissue (taken from the resection margin of the CRC test set; n = 3) samples using Trizol (Invitrogen, Carlsbad, CA, USA). RNA from 16 additional colorectal cancer samples (CRC test set) was extracted using AllPrep DNA/RNA Mini Kit (Qiagen). The RNA quality was measured using a 2100 Bioanalyzer (Agilent Technologies, Santa Clara, CA, USA) and the concentration was determined using ND-1000 Nanodrop (NanoDrop Technologies, Wilmington, DE, USA).

Total RNA was converted to cDNA using the High-Capacity cDNA Archive kit (Applied Biosystems), including random primers. The cDNA of *CNRIP1 *(Hs00384403_m1), *FBN1 *(Hs00171191_m1), *INA *(Hs00190771_m1), *SNCA *(Hs00240906_m1), and the endogenous controls *ACTB *(Hs99999903_m1) and *GUSB *(Hs99999908_m1) was amplified separately in 384 well plates according to the manufacturers' protocol (Applied Biosystems), and the resulting quantitative gene expression measurements were registered by the 7900HT Sequence Detection System (Applied Biosystems). Samples were analyzed in triplicates, and the median value was used for data analysis. The human universal reference RNA (containing a mixture of total RNA from ten different cell lines; Agilent) was used to generate a standard curve, and the resulting quantitative expression levels of *CNRIP1, FBN1, INA*, and *SNCA *were normalized against the mean value of the two endogenous controls.

### Ethics

According to National legislation all samples belong to approved research biobanks and approvals are given by the Regional Ethics Committee (S-09282c2009/4958 biobank 2781;S95151).

### Statistical analysis

For statistical analyses, SPSS 16.0 (SPSS, Chicago, IL, USA) was used. Pearson's chi-square and Fisher's exact tests were used for categorical variables. Student T-test and Mann-Whitney U test were used to investigate potential associations between tumor DNA methylation and patient age and polyp size. Student T-test was also used to examine the relationship of aberrant promoter methylation to gene expression in tissue samples and cancer cell lines. All *P *values derive from two-tailed tests. Receiver Operating Characteristics (ROC) curves for individual biomarkers were generated using percentage methylated reference (PMR) values and tissue type (cancer or adenoma and normal) as input. For evaluation of the combined biomarker panel the sum of PMR values from *CNRIP1, FBN1, INA, MAL, SNCA*, and *SPG20 *was used.

## Results

### Identification of the most suitable biomarkers

**Figure 2:**
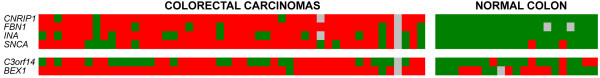
**Summary of promoter methylation status in test sets of colorectal cancer and normal colorectal tissue**. Red color indicates methylated sample, green color indicates unmethylated sample, and grey color indicates samples that were not successfully amplified and thereby not scorable. Gene promoters in the upper panel (*CNRIP1, FBN1, INA *and *SNCA*) were methylated in 10% or less of the normal tissue samples tested and represent biomarkers with potentially high specificities, and thus suitable for a future diagnostic test. Gene promoters in the lower panel (*C3orf14 *and *BEX1*) were methylated more frequently than 10% in normal tissue samples, which might limit the specificity in a test situation, and were excluded from further analyses.

In the evaluated 20 colon cancer cell lines, all but one (*UBE3A*) gene promoters were hypermethylated with frequencies ranging from 20% (*LEF1 *and *MEF2C*) to 100% (*CNRIP1*; Figure 1, and Additional file 2, Figure S1). Six genes (*BEX1, C3orf14, CNRIP1, FBN1, INA*, and *SNCA*) were methylated in > 80% of the cell lines, representing promising biomarkers in terms of sensitivity. These were subjected to further detailed analyses in parts of the colorectal cancer (n = 51) and normal tissue (n = 21) test sets using qualitative MSP analysis. As shown in Figure 2, for all genes the frequency of promoter methylation was significantly higher in cancers compared to the normal tissue samples. Methylation in colorectal cancers ranged from 55% (*C3orf14*) to 96% (*BEX1*; median 84%), while methylation in normal tissue was between 0% (*CNRIP1, FBN1, INA*) and 45% (*BEX1*; median 5%), *P *< 0.0001 to *P *< 0.02. To increase the likelihood of identifying biomarkers with tumor specific methylation, only gene promoters with methylation frequencies equal to or lower than 10% in normal samples (*CNRIP1, FBN1, INA*, and *SNCA*) were subjected to further analyses.

### Quantitative DNA methylation analyses in test sets: cancers, adenomas, and normal colorectal samples

*CNRIP1, FBN1, INA, MAL*, and *SNCA *promoter methylation was analyzed quantitatively (qMSP) in the colorectal cancer (n = 74; median age 71 years) and normal mucosa (n = 51; median age 55 years) test sets. We found overall methylation percentages of 99, 81, 66, 92, and 73 in colorectal cancers and 2, 2, 0, 2, and 0 in normal mucosa, respectively (Table 2). Quantitative data for *SPG20 *[9] was included in a biomarker panel evaluation. Co-methylation, here defined as simultaneous hypermethylation of two or more of the six gene promoters (*CNRIP1, FBN1, INA, MAL, SNCA*, and *SPG20*), was found in 99% of the colorectal cancer test set samples and 2% of the normal mucosa samples. ROC curves of the six biomarkers combined resulted in an area under the ROC curve (AUC) value of 0.999 (Additional file 1, Table S3).

**Table 2:** Methylation frequencies in the analyzed sample cohorts assessed by quantitative methylation-specific polymerase chain reaction

Samples/Biomarkers	*CNRIP1*	*FBN1*	*INA*	*SNCA*	*MAL*	*SPG20^§^*	Biomarker panel^†^
Colon Cancer Cell Lines*	20/20 (100%)	18/20 (90%)	19/20 (95%)	18/20 (90%)	19/20 (95%)	20/20 (100%)	20/20 (100%)
Adenomas Test Set	54/60 (90%)	41/60 (68%)	25/60 (42%)	33/60 (55%)	51/60 (85%)	45/60 (75%)	54/60 (90%)
Adenomas Validation Set	47/51 (92%)	36/51 (71%)	14/51 (27%)	26/51 (51%)	42/51 (82%)	42/51 (82%)	49/51 (96%)
**Adenomas Combined (Test and Validation Sets)**	**101/111 (91%)**	**77/111 (69%)**	**39/111 (35%)**	**59/111 (53%)**	**93/111 (84%)**	**87/111 (78%)**	**103/111 (93%)**

CRC Test Set	73/74 (99%)	60/74 (81%)	49/74 (66%)	54/74 (73%)	68/74 (92%)	67/74 (91%)	73/74 (99%)
CRC Validation Set	96/105 (91%)	82/105 (78%)	68/105 (65%)	65/105 (62%)	94/105 (90%)	92/105 (88%)	95/105 (90%)

Normal Mucosa Matching CRC Validation Set	42/105 (40%)	2/105 (2%)	0/105 (0%)	0/105 (0%)	3/105 (3%)	0/105 (0%)	4/105 (4%)
**CRCs Combined (Test and Validation Sets)**	**169/179 (94%)**	**142/179 (79%)**	**117/179 (65%)**	**119/179 (66%)**	**162/179 (91%)**	**159/179 (89%)**	**168/179 (94%)**

Normal Mucosa Test Set	1/51 (2%)	1/51 (2%)	0/51 (0%)	0/51 (0%)	1/51 (2%)	1/51 (2%)	1/51 (2%)
Normal Mucosa Validation Set	5/59 (8%)	0/59 (0%)	0/59 (0%)	0/59 (0%)	1/59 (2%)	0/59 (0%)	1/59 (2%)
**Normals Combined (Test and Validation Sets)**	**6/110 (5%)**	**1/110 (1%)**	**0/110 (0%)**	**0/110 (0%)**	**2/110 (2%)**	**1/110 (1%)**	**2/110 (2%)**

Abbreviation: CRC, colorectal cancer.

* Colon Cancer Cell lines have been analyzed using qualitative methylation-specific PCR.

^§ ^Previously published results [9].

† Simultaneous methylation of two or more of the six markers provides a positive biomarker panel.

Within 60 adenoma samples (median age 67 years) successfully amplified by qMSP, promoter hypermethylation was detected in 90%, 68%, 42%, 85% and 55% for *CNRIP1, FBN1, INA, MAL*, and *SNCA*, respectively, and 90% of the adenomas harbored co-methylation. ROC curves of the combined biomarker panel gave an AUC value of 0.981 in adenomas versus normal mucosa test set (Additional file 1, Table S3). The 105 normal mucosa samples (median age 71 years) taken from the resection margins of the cancer specimens in the colorectal cancer validation set showed promoter methylation in 40%, 2%, 0%, 3%, and 0% of the same genes, and 4% harbored co-methylation.

### DNA methylation analyses in validation series

**Figure 3:**
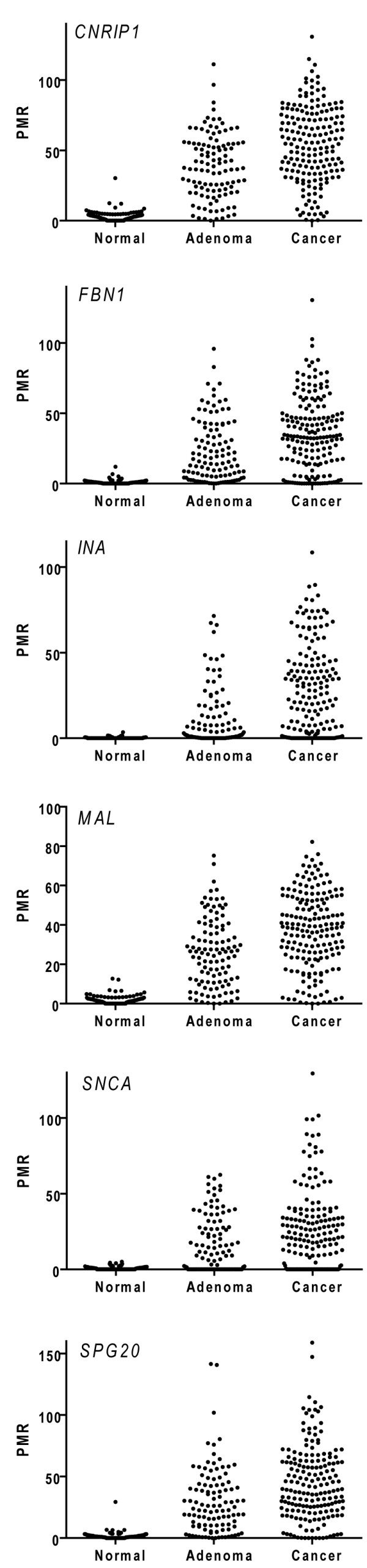
**Percent methylated reference (PMR) values of all biomarkers in combined test and validation sets of normal mucosa, adenomas, and colorectal cancer**. Note that for *SPG20 *two outliers (PMR > 150) are excluded from the graph. The *SPG20 *promoter methylation status has previously been published in the same sample series [9].

The frequencies of methylation in the validation series were comparable with the findings in the test set (Table 2). Ninety-five out of 105 colorectal cancers (90%; median age 71 years) and 49 out of 51 adenomas (96%; median age 58 years) were hypermethylated in at least two of the six analyzed markers, in contrast to one of the 59 normal mucosa samples (2%; median age 67 years). The distribution of PMR values in the normal mucosa samples, the adenomas, and cancers is illustrated in Figure 3. The AUC values from the ROC analysis were 0.963 for colorectal cancers versus normal mucosa and 0.962 for adenomas versus normal mucosa (Additional file 1, Table S3).

### Associations of genetic and clinico-pathologic data with tumor methylation of individual genes

Across the test (n = 74) and validation (n = 105) series, 33 out of 179 (18%) colorectal cancers harbored a *BRAF *mutation in exon 15. In 23 out of 48 colorectal cancer cases with microsatellite instability (MSI-high; 48%), a *BRAF *mutation in exon 15 was present, compared to only 10 out of 131 (8%) of the MSS/MSI-low tumors (*P *= 8.9E-9).

Methylation of the individual genes was more common among proximal, MSI-high, and *BRAF *mutated cancers, although not statistically significant for all comparisons. DNA methylation of each gene was equally frequent in colorectal cancers of all stages (I-IV) as well as in tumors from female and male patients (Table 3). The age of patients with an *INA *methylation positive cancer was slightly higher (mean 72 years) than that of patients with an *INA *methylation negative cancer (mean 65 years; *P *= 1.4E-4; T-test). However, all normal colorectal samples tested (n = 215) contained unmethylated *INA *promoters, ruling out age-specific methylation.

**Table 3:** Promoter hypermethylation of biomarkers in colorectal carcinomas (test and validation sets) compared with the patients clinico-pathological features and tumor phenotype

	*CNRIP1*		*FBN1*		*INA*		*MAL*		*SNCA*		Panel	
	
	M	U	M	U	M	U	M	U	M	U	Pos	Neg
**Tumors**												
No	169/179	10/179	142/179	37/179	117/179	62/179	162/179	17/179	119/179	60/179	168/179	11/179
**Tumor phenotype**												
MSI	47	1	42	6	36	11	46	2	37	10	47	1
MSS	122	9	100	31	81	51	116	15	82	50	121	10
*P *value	NS		NS		7.4E-2		NS		7.6E-2		NS	
***BRAF *ex15**												
Wild Type	136	10	109	37	87	59	129	17	89	57	135	11
Mutation	33	0	33	0	30	3	33	0	30	3	33	0
*P value*	NS		**2.5E-4**		**4.5E-4**		**3.9E-2**		**8.5E-4**		NS	
**Sex**												
Male	83	6	71	18	57	32	81	8	57	32	81	8
Female	86	4	71	19	60	30	81	9	62	28	87	3
*P *value	NS		NS		NS		NS		NS		NS	
**Tumor site - 2 groups**												
Proximal	67	3	57	13	53	17	65	5	50	20	66	4
Distal	100	7	83	24	63	44	95	12	67	40	100	7
*P *value	NS		NS		**2.4E-2**		NS		NS		NS	
**Tumor site - 3 groups**												
Right	67	3	57	13	53	17	65	5	50	20	66	4
Left	57	0	48	9	36	21	54	3	39	18	57	0
Rectum	43	7	35	15	27	23	41	9	28	22	43	7
*P *value	**6.1E-3**		NS		**4.3E-2**		5.5E-2		NS		**1.1E-2**	
**Stage**												
I	27	3	23	7	19	11	26	4	20	10	27	3
II	76	3	60	19	54	25	72	7	52	27	75	4
III	45	4	42	7	31	18	44	5	33	16	45	4
IV	21	0	17	4	13	8	20	1	14	7	21	0
*P *value	NS		NS		NS		NS		NS		NS	

Pearson's chi-square and Fisher's exact tests were used to calculate *P*-values. Associations between *SPG20 *promoter hypermethylation and tumor phenotype have been published elsewhere [9].

Abbreviations: MSI, microsatellite instability; MSS, microsatellite stable; Neg, biomarker panel negative; NS, not significant; Pos, biomarker panel positive. A positive biomarker panel is defined as methylation of two or more of the following biomarkers: *CNRIP1, FBN1, INA, MAL, SNCA *and *SPG20*.

Among the adenomas, *INA *and *FBN1 *methylation was more frequently found in large (10 mm or larger) than in small adenomas (smaller than 10 mm in diameter; *P *= 0.012 and *P *= 0.047, respectively; T-test). Additional detailed information about clinical associations is presented in Table 4.

**Table 4:** Promoter hypermethylation of biomarkers in colorectal adenomas (test and validation sets) compared with the patients clinico-pathological features and tumor phenotype

	*CNRIP1*		*FBN1*		*INA*		*MAL*		*SNCA*		Panel	
	
	M	U	M	U	M	U	M	U	M	U	Pos	Neg
**Tumors**												
No	101/111	10/111	77/111	34/111	39/111	72/111	93/111	18/111	59/111	52/111	103/111	8/111
**Tumor phenotype**												
MSI	2	0	2	0	1	1	2	0	1	1	2	0
MSS	52	6	39	19	24	34	49	9	32	26	52	6
*P *value	NS		NS		NS		NS		NS		NS	
**Sex**												
Male	50	4	41	13	22	32	46	8	31	23	51	3
Female	51	6	36	21	17	40	47	10	28	29	52	5
*P *value	NS		NS		NS		NS		NS		NS	
**Tumor site - 2 groups**												
Proximal	15	2	11	6	5	12	14	3	9	8	15	2
Distal	85	7	64	28	33	59	77	15	50	42	86	6
*P *value	NS		NS		NS		NS		NS		NS	
**Tumor site - 3 groups**												
Right	15	2	11	6	5	12	14	3	9	8	15	2
Left	54	6	37	23	17	43	49	11	31	29	55	5
Rectum	31	1	27	5	16	16	28	4	19	13	31	1
*P *value	NS		7.5E-2		NS		NS		NS		NS	
**Dysplasia**												
High	7	0	5	2	2	5	7	0	5	2	7	0
Low	94	10	72	32	37	67	86	18	54	50	96	8
*P *value	NS		NS		NS		NS		NS		NS	
**Tumor size**												
< 10 mm	45	2	31	16	12	35	39	8	23	24	44	3
≥ 10 mm	55	7	44	18	26	36	52	10	36	26	57	5
*P *value	NS		NS		NS		NS		NS		NS	

Pearson's chi-square and Fisher's exact tests were used to calculate *P*-values. Associations between *SPG20 *promoter hypermethylation and tumor phenotype have been published elsewhere [9].

Abbreviations: MSI, microsatellite instability; MSS, microsatellite stable; Neg, biomarker panel negative; NS, not significant; Pos, biomarker panel positive. A positive biomarker panel is defined as methylation of two or more of the following biomarkers: *CNRIP1, FBN1, INA, MAL, SNCA *and *SPG20*.

### Associations of genetic and clinico-pathologic tumor sample data with a combined biomarker panel

**Figure 4:**
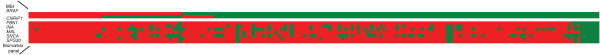
**Summary of genetic and epigenetic findings in colorectal cancers (test and validation sets)**. Red color: methylated (*CNRIP1, FBN1, INA, MAL, SNCA*, and/or *SPG20*), mutated (*BRAF*), and/or MSI positive samples. Green color: unmethylated, wt (*BRAF*), and/or MSS/MSI-low samples. Biomarker panel positive samples have co-methylation of two or more of the six biomarkers. The *SPG20 *promoter methylation status has previously been published in the same sample series [9].

**Figure 5:**
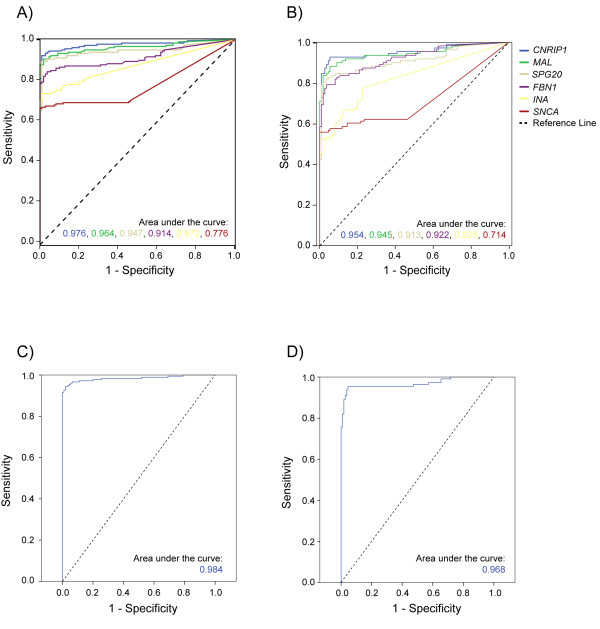
**Receiver Operating Characteristics (ROC) curves for methylation of individual and combined biomarkers in colorectal cancers and adenomas versus normal mucosa (test and validation sets)**. The area under the ROC curve (AUC) signifies the accuracy of the individual and combined biomarkers for distinguishing colorectal cancers (A and C) and adenomas (B and D) from normal colorectal tissue samples. A) Colorectal cancers versus controls for individual biomarkers. B) Adenomas versus controls for individual biomarkers. All six biomarkers are combined in C) Colorectal cancers versus controls and D) adenomas versus controls. Asymptotic significance, standard error and 95% confidence interval measurements for all values can be found in Additional file 1, Table S3. The ROC curves for the *SPG20 *biomarker has been published previously in the same sample series [9].

Co-methylation of two out of six genes (*CNRIP1, FBN1, INA, MAL, SNCA*, and *SPG20*) was not associated with patient gender, and neither with cancer stage, proximal or distal location, nor *BRAF *and MSI status (Table 3). A Mann-Whitney U analysis showed a non-significant trend (*P *= 0.067) towards higher age among colorectal cancer patients positive for the biomarker panel compared with negative patients, but this was not confirmed in the other sample series (adenomas, normal mucosa, and normal mucosa from cancer patients). All colorectal cancers that harbored *BRAF *mutation as well as the vast majority (98%) of cancers with the MSI phenotype were biomarker panel positive, in line with the CIMP concept [34]. The biomarker panel was additionally positive in 111/121 (92%) cancers harboring the MSS/MSI-low phenotypes and wild-type *BRAF *(Figure 4). Cancers located in the colon, and particularly the distal colon, showed somewhat more frequent co-methylation than did cancers located in the rectum (*P *= 0.011). The combined biomarker panel reached an AUC value of 0.984 across the colorectal cancer validation and test sets (*P *= 1.9E-43; Figure 5; Additional file 1, Table S3).

The biomarker panel was also positive in adenomas independent of clinico-pathological characteristics as there was no significant association between positive tumors and MSI status (only two MSI positive), tumor location, polyp size, or patient age or sex (Table 4). For adenomas, the AUC value of the combined test and validation sets was 0.968 (*P *= 2.6E-33; Figure 5; Additional file 1, Table S3).

### Validation of promoter methylation status by direct bisulfite sequencing

**Figure 6:**
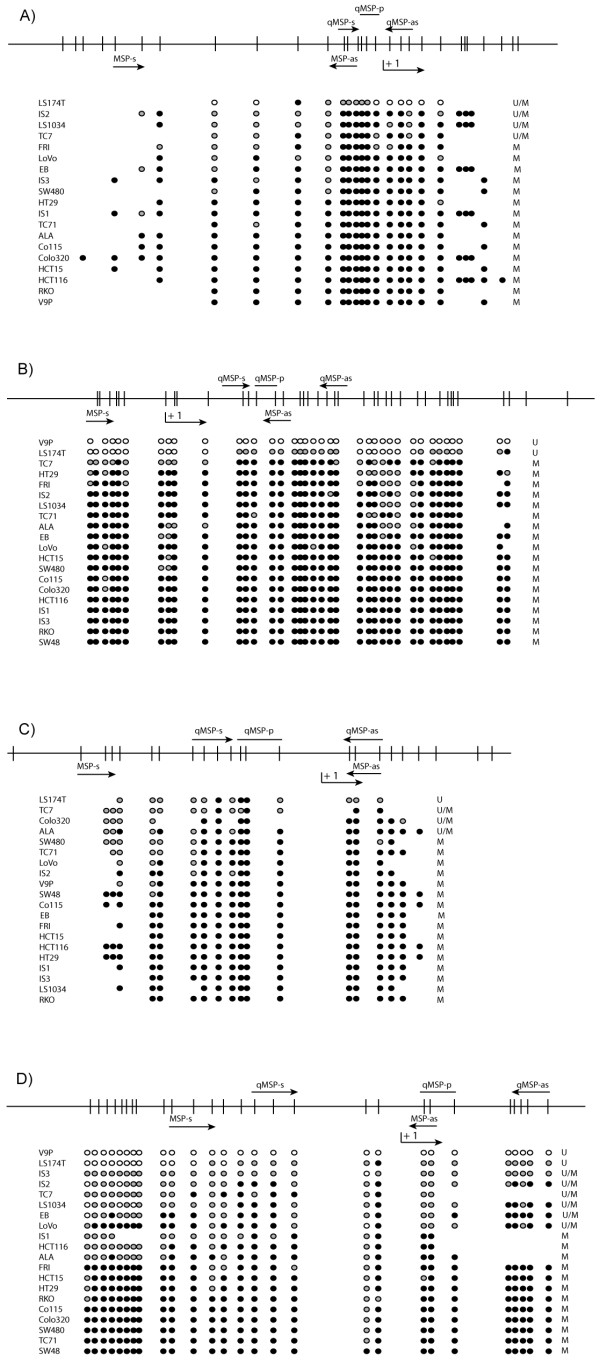
**Direct bisulfite sequencing of *CNRIP1, FBN1, INA*, and *SNCA *confirmed methylation status as assessed by methylation-specific polymerase chain reaction (MSP)**. A) *CNRIP1*. B) *FBN1*. C) *INA*. D) *SNCA*. For all panels, the upper part is a schematic presentation of the CpG sites (vertical bars) amplified by the bisulfite sequencing primers (110 to 470, NM_015463; -85 to 325, NM_000138; -110 to 71, NM_032727; and -169 to 91, NM_000345, respectively). The transcription start site is represented by +1 and the arrows indicate the location of the MSP and qMSP primers. For the lower part of the panels, black circles represent methylated CpGs (the presence of more than 80% cytosine); white circles represent unmethylated CpGs (0 to 20% cytosine); and gray circles represent partially methylated sites (20-80% cytosine). The column of U, M, and U/M at the right side of this lower part lists the methylation status of the respective cell lines as assessed by us using MSP analyses. The transcription start point of *CNRIP1 *is here according to hg17 NM_015463. When the gene was annotated the transcription start point was moved 340 bases upstream of the indicated position. Abbreviations: MSP, methylation-specific polymerase chain reaction; s, sense; as, antisense; p, probe; U, unmethylated; M, methylated; U/M, presence of both unmethylated and methylated band.

Methylation status of representative samples was confirmed by direct bisulfite sequencing using primers that flank the MSP and qMSP regions of *CNRIP1, FBN1, INA*, and *SNCA *(Figure 6).

### Promoter methylation in relation to gene expression

**Figure 7:**
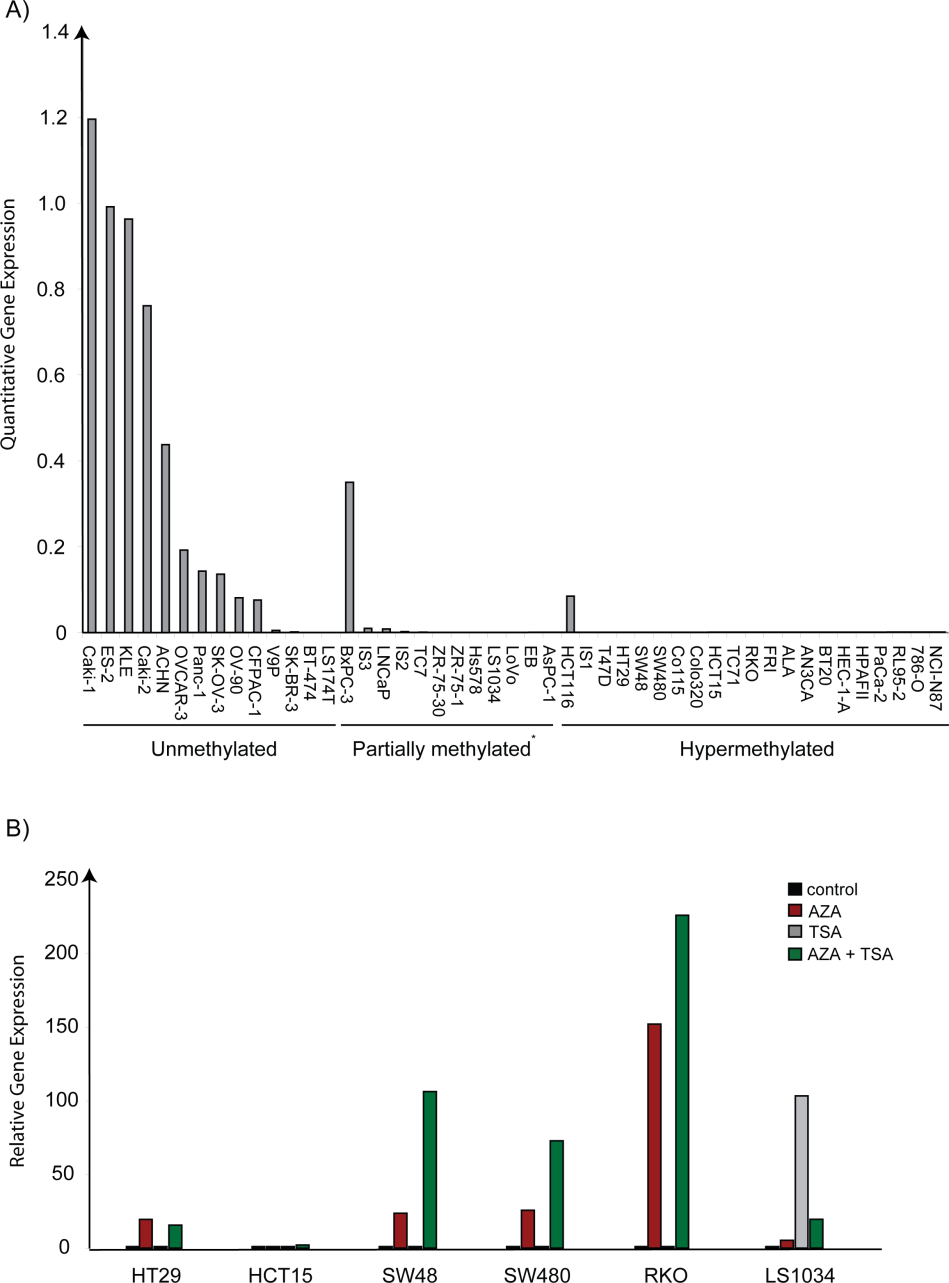
**Real-time PCR analysis of *SNCA *in cancer cell lines with known methylation status**. A) Gene expression and DNA promoter methylation status of *SNCA *in cancer cell lines. The quantitative gene expression levels are displayed as ratios between the median of *SNCA *and the average of two endogenous controls, *GUSB *and *ACTB*. *Partially methylated signifies cell lines with the presence of both a methylated and unmethylated band for the MSP analysis, which most likely reflect monoallelic or heterogeneous methylation. B) Relative gene expression of *SNCA *in six colon cancer cell lines treated with 1 μM of 5-aza-2'deoxycytidine for 72 h (AZA), 0.5 μM of trichostatin A for 12 h (TSA), and a combination of both drugs (AZA+TSA).

In order to examine the relationship of aberrant promoter methylation to gene expression, cancer cell lines, colorectal cancers, and normal mucosa samples were subjected to quantitative real time analysis. The methylation status of *CNRIP1, FBN1, INA*, and *SNCA *across all cancer cell lines (n = 49) can be seen in Additional file1, Figure S1. The level of mRNA expression of *CNRIP1, FBN1, INA*, and *SNCA *was strongly associated with promoter methylation status in cancer cell lines (*P *= 0.037, *P *= 0.017, *P *= 0.006, and *P *= 0.001, respectively; Figure 7A).

Analysis of tissue samples supported that DNA promoter methylation was significantly associated with reduced gene expression for *CNRIP1, INA*, and *SNCA *(Additional file 1, Table S4). The mean mRNA expression of *FBN1 *in methylated samples was lower than in unmethylated samples (0.4 versus 0.8), although this was not statistically significant (*P *= 0.125)

An association between promoter DNA methylation and gene expression was further confirmed by measurements of mRNA levels in colon cancer cell lines treated with epigenetic drugs (both 5-aza-2'deoxycytidine and trichostatin A, alone and in combination). Before drug treatment all colon cancer cell lines tested harbored promoter methylation of *CNRIP1, INA, FBN1*, and *SNCA *accompanied by little or no expression of the same genes. With the exception of *FBN1*, the mRNA levels of all genes subjected to real-time PCR analysis were up-regulated in a least four of the six treated cell lines (Figure 7B). The combined results from real-time analyses using both *in vivo *as well as *in vitro *samples thereby suggest that *CNRIP1, INA*, and *SNCA *gene expression might be subjected to epigenetic regulation.

## Discussion

We have identified four genes, *CNRIP1, FBN1, INA*, and *SNCA *that were frequently hypermethylated in colorectal adenomas and cancers. The methylation was highly tumor-specific, as only a minority of normal mucosa samples harbored promoter hypermethylation of the same genes, fulfilling the criteria for cancer-specific methylation (type C) defined by Toyota and co-workers [34]. These genes in combination with the previously reported *MAL *[21,22] and *SPG20 *[9] resulted in a biomarker panel with a sensitivity of 94% for colorectal cancers and 93% for adenomas, and a specificity of 98%. A high specificity will increase the positive predictive value in screening and thereby limit the number of false positives and subsequent unnecessary colonoscopies.

The use of genome-wide experimental approaches for identification of novel target genes for tumor-specific DNA methylation has provided long candidate gene lists [35-38], and a substantial number of genes has been analyzed in detail, *i.e*. [36,39,40]. In spite of this, only few genes, including *GALR2, ITGA4, NTRK2, OSMR, SFRP1, SFRP2, SLC16A12, TUBG2, MAL *and *NMDAR2A *[8,14,21,22,36,41-43] (reviewed in [44] and [15]) have been found to harbor promoter methylation in more than 80% of colorectal cancers analyzed, and simultaneously in less than 10% of normal mucosa samples, opening up the possibility of achieving high sensitivities and specificities in a future test. Up to now, only a few of the markers mentioned above have been analyzed in non-invasive sample material [13,14,42,45-47]. Additionally, the promising biomarkers *SEPT9 *[48] and *VIM *[12] have been analyzed in blood/serum samples and stool samples, respectively and are the only markers included in currently available non-invasive tests. Vimentin is the target gene of the ColoSure™ test, which has a company-reported sensitivity and specificity range of 72-77% and 83-94%, respectively. *SEPT9 *is the target gene of the plasma based Epi *Pro*Colon early detection assay which can detect 68%-72% of colorectal cancers with a specificity of 89%-93% [48]. Despite that the first test analyzes stool samples and the second test is blood based, the sensitivity and specificity measurements are quite comparable, both leaving room for improvements. This is underscored by an independent report finding vimentin methylation in only 72% of colorectal cancers and in as much as 11% of normal mucosa samples [39]. In the present study, four of the biomarkers (*CNRIP1, FBN1, MAL*, and *SPG20*) were more frequently methylated in colorectal cancers than previously reported for the vimentin gene, and additionally less frequently methylated in normal mucosa samples, underscoring the suitability of these markers for diagnostic use. The most important results in the present study is, however, the high performance of the combined biomarker panel and the robustness such a panel offers in a future test compared to single markers.

As expected, the identified methylation markers were frequently detected in MSI positive colorectal cancers containing *BRAF *mutations, features that are consistent with the CIMP positive phenotype [34]. However, due to the broad coverage of the present biomarker panel, not only cancers harboring these features were biomarker positive, but also the majority of MSS tumors with wild-type *BRAF*. It is essential that a diagnostic biomarker panel adequately "represents" the entire large bowel, in order to detect all pheno- and geno-typically different colorectal tumors. The present biomarker panel is positive in tumors independent of the patient's gender and age, as well as of tumor stage and location. Even though tumors of all clinico-pathological subtypes were represented among the biomarker panel methylation positive samples, the very few negative colorectal cancers were generally of the MSS phenotype and were *BRAF *wild-type, compatible with CIMP negative tumors [34]. However, it should be noted that in the present study we have used the test sets to establish the most optimal threshold for each biomarker assay (resulting in the highest specificity). From these values, we chose the highest and most conservative scoring threshold (percentage methylated sample - PMR value) and applied this to all biomarkers. Alternatively, by setting the threshold for each individual gene, the sensitivity could be increased without necessarily affecting the specificity.

In spite of several genome-wide epigenetic studies, only a few reports include DNA methylation data and potential subsequent epigenetic silencing of *CNRIP1, FBN1, INA*, and *SNCA *in cancer [10,49-53]. Due to the exceptionally high methylation frequencies of these genes in colorectal tumors they are suitable as biomarkers, but the potential roles of the encoded proteins in tumor genesis remain unknown. Interestingly, mutations in *FBN1*, which is a member of the fibrillin family, are associated with the Marfan syndrome [49]. *FBN1 *methylation has previously been identified in prostate cancer cell lines [50] and the gene has also been shown to be epigenetically silenced in tumor endothelial cells [51]. Functional validation by RNA interference in endothelial cells pinpointed FBN1 as a negative regulator of cell growth and angiogenesis [51]. The expression of the synuclein alpha gene, *SNCA*, was recently shown to be regulated by methylation and decreased in the brain tissue of patients with Parkinsons's disease [52]. The gene has also been reported methylated in 38% of breast cancer tumors [53]. Interestingly, the majority of adjacent normal tissue samples were also found to be methylated, indicating a potential field defect that could help pinpointing geographical zones of increased breast cancer risk [53]. Such a field defect was not seen in the present study for *SNCA*, where all normal mucosa samples taken in distance from the colorectal cancers were unmethylated. In contrast, we observed a 40% promoter methylation frequency of *CNRIP1 *in the same sample group. The majority of normal mucosa samples from colorectal cancer free individuals (95%) were unmethylated in the *CNRIP1 *gene promoter. The methylation positive normal mucosa cells adjacent to the methylated cancer could potentially increase the detection of this aberration in stool samples, by increasing the total number of methylation positive cells shed into the lumen. In 2006 we identified *CNRIP1 *as a promoter methylation target in colorectal cancer by treatment of cell lines with 5-aza 2'deoxycytidine followed by expression microarray analyses [20,54]. By applying DNA methylation microarray analysis and subsequent methylation-sensitive high resolution melting analysis colorectal tumor specific promoter methylation of *CNRIP1 *was recently confirmed [10]. Most of the adenomas (n = 12) and carcinomas (n = 55) analyzed were methylated [10] in agreement with the present report, underscoring the potential of *CNRIP1 *as a novel marker for early detection of colorectal tumors.

Here we found that the DNA methylation of *CNRIP1, FBN1, INA*, and *SNCA *was associated with reduced or lost gene expression in cell lines, indicating that they might harbor a tumor suppressor function. Down-regulation in colorectal cancers compared with normal mucosa was also seen for all genes from *in silico *analyses, including *MAL *and *SPG20*, using the GeneSapiens website, which contains Affymetrix gene expression array experiments representing more than 500 colorectal cancers and 23 normal colon samples [55].

## Conclusions

In the present study we identified and validated four novel methylated genes with high sensitivity and specificity for both colorectal cancers and adenomas. A combination of these and two of our previously identified biomarkers (*MAL *[21,22] and *SPG20 *[9]) provided an excellent biomarker panel. Early detection of colorectal cancer, at a stage where it is localized and curable will contribute substantially to reduce mortality due to the disease. Moreover, the present biomarker panel has been shown to also be positive in premalignant adenomas. Therefore, a test using this panel may also provide non-invasive detection of lesions prior to malignancy, thereby potentially reducing the incidence of colorectal cancer. The advantage of a marker panel compared to single marker analysis is obviously the higher sensitivity and specificity but also increased robustness. A single marker will be more prone to biological and/or technical failure than a combined panel. Finally, the benefit of a non-invasive epigenetic test compared to a genetic test is the simplicity of performance, which is expected to lower the costs, hence supporting it as appropriate for screening purposes. The presented biomarker panel seems highly suitable for development of a non-invasive test for early detection of colorectal tumors.

## List of abbreviations

AUC: area under the curve; MSI: microsatellite instability; MSP: methylation-specific polymerase chain reaction; MSS: microsatellite stable; qMSP: quantitative methylation-specific polymerase chain reaction; ROC: receiver operating characteristics.

## Authors' contributions

GEL, SAD, TA, KA, and MH carried out the experimental analyses. GEL, SAD and RIS carried out data interpretation and statistical analyses. GEL contributed to the study design and drafted the manuscript. MAM, TOR, GIM, GH, MB, ETE, and AN collected clinical samples and provided patient data. RAL conceived the study and contributed to its design, and participated in interpretation of results, and in manuscript preparation. All authors have read and approved the final manuscript.

## Supplementary Material

Additional file 1 **Supplemental tables**. Click here for file

Additional file 2 **Figure S1. Summary of methylation-specific polymerase chain reaction (MSP) results in cancer cell lines**. Red color indicates methylated sample, orange color indicates the presence of both methylated and unmethylated bands, green color indicates unmethylated sample, and grey color indicates samples that were not successfully amplified/analyzed. The upper part of the figure illustrates the results from colon cancer cell lines (n = 20) and the lower part of the figure from cell lines originating from various cancer tissues (n = 29). Click here for file

## References

[B1] Jemal A Siegel R Ward E Hao Y Xu J Murray T Thun MJ Cancer statistics, 2008 CA Cancer J Clin 2008 58 71 96 10.3322/CA.2007.0010 18287387

[B2] Levin B Lieberman DA McFarland B Andrews KS Brooks D Bond J Dash C Giardiello FM Glick S Johnson CD Levin TR Pickhardt PJ Rex DK Smith RA Thorson A Winawer SJ Screening and surveillance for the early detection of colorectal cancer and adenomatous polyps, 2008: a joint guideline from the American Cancer Society, the US Multi-Society Task Force on Colorectal Cancer, and the American College of Radiology Gastroenterology 2008 134 1570 1595 10.1053/j.gastro.2008.02.002 18384785

[B3] Bretthauer M Evidence for colorectal cancer screening Best Pract Res Clin Gastroenterol 2010 24 417 425 10.1016/j.bpg.2010.06.005 20833346

[B4] Atkin WS Edwards R Kralj-Hans I Wooldrage K Hart AR Northover JM Parkin DM Wardle J Duffy SW Cuzick J Once-only flexible sigmoidoscopy screening in prevention of colorectal cancer: a multicentre randomised controlled trial Lancet 2010 375 1624 1633 10.1016/S0140-6736(10)60551-X 20430429

[B5] van DL Kuipers EJ van Leerdam ME Performance improvements of stool-based screening tests Best Pract Res Clin Gastroenterol 2010 24 479 492 10.1016/j.bpg.2010.03.009 20833351

[B6] Rashid A Shen L Morris JS Issa JP Hamilton SR CpG island methylation in colorectal adenomas Am J Pathol 2001 159 1129 1135 10.1016/S0002-9440(10)61789-0 11549606 PMC1850474

[B7] Chan AO Broaddus RR Houlihan PS Issa JP Hamilton SR Rashid A CpG island methylation in aberrant crypt foci of the colorectum Am J Pathol 2002 160 1823 1830 10.1016/S0002-9440(10)61128-5 12000733 PMC1850869

[B8] Chang E Park DI Kim YJ Kim BK Park JH Kim HJ Cho YK Sohn CI Jeon WK Kim BI Kim HD Kim DH Kim YH Detection of colorectal neoplasm using promoter methylation of ITGA4, SFRP2, and p16 in stool samples: a preliminary report in Korean patients Hepatogastroenterology 2010 57 720 727 21033217

[B9] Lind GE Raiborg C Danielsen SA Rognum TO Thiis-Evensen E Hoff G Nesbakken A Stenmark H Lothe RA SPG20, a novel biomarker in colorectal carcinogenesis, encodes a regulator of cytokinesis Oncogene 2011 1 12 10.1038/onc.2011.109 PMC3174365 21499309

[B10] Øster B Thorsen K Lamy P Wojdacz TK Hansen LL Birkenkamp-Demtrõder K Sørensen KD Laurberg S Ørntoft TF Andersen CL Identification and validation of highly frequent CpG island hypermethylation in colorectal adenomas and carcinomas Int J Cancer 2011 10 10.1002/ijc.25951 21400501

[B11] Müller HM Oberwalder M Fiegl H Morandell M Goebel G Zitt M Mühlthaler M Öfner D Margreiter R Widschwendter M Methylation changes in faecal DNA: a marker for colorectal cancer screening? Lancet 2004 363 1283 1285 10.1016/S0140-6736(04)16002-9 15094274

[B12] Chen WD Han ZJ Skoletsky J Olson J Sah J Myeroff L Platzer P Lu S Dawson D Willis J Pretlow TP Lutterbaugh J Kasheri L Willson JK Rao JS Shuber A Markowitz SD Detection in fecal DNA of colon cancer-specific methylation of the nonexpressed vimentin gene J Natl Cancer Inst 2005 97 1124 1132 10.1093/jnci/dji204 16077070

[B13] Zhang W Bauer M Croner RS Pelz JO Lodygin D Hermeking H Sturzl M Hohenberger W Matzel KE DNA Stool Test for Colorectal Cancer: Hypermethylation of the Secreted Frizzled-Related Protein-1 Gene Dis Colon Rectum 2007 50 1618 1626 10.1007/s10350-007-0286-6 17762966

[B14] Kim MS Louwagie J Carvalho B Terhaar Sive Droste JS Park HL Chae YK Yamashita K Liu J Ostrow KL Ling S Guerrero-Preston R Demokan S Yalniz Z Dalay N Meijer GA van Criekinge W Sidransky D Promoter DNA methylation of oncostatin m receptor-beta as a novel diagnostic and therapeutic marker in colon cancer PLoS ONE 2009 4 e6555 10.1371/journal.pone.0006555 19662090 PMC2717211

[B15] Kim MS Lee J Sidransky D DNA methylation markers in colorectal cancer Cancer Metastasis Rev 2010 29 181 206 10.1007/s10555-010-9207-6 20135198

[B16] Tang D Liu J Wang DR Yu HF Li YK Zhang JQ Diagnostic and prognostic value of the methylation status of secreted frizzled-related protein 2 in colorectal cancer Clin Invest Med 2011 34 E88 E95 21463549 10.25011/cim.v34i1.15105

[B17] Herbst A Rahmig K Stieber P Philipp A Jung A Ofner A Crispin A Neumann J Lamerz R Kolligs FT Methylation of NEUROG1 in Serum Is a Sensitive Marker for the Detection of Early Colorectal Cancer Am J Gastroenterol 2011 106 1110 1118 10.1038/ajg.2011.6 21326223

[B18] Tänzer M Balluff B Distler J Hale K Leodolter A Rocken C Molnar B Schmid R Lofton-Day C Schuster T Ebert MP Performance of epigenetic markers SEPT9 and ALX4 in plasma for detection of colorectal precancerous lesions PLoS ONE 2010 5 e9061 10.1371/journal.pone.0009061 20140221 PMC2816214

[B19] He Q Chen HY Bai EQ Luo YX Fu RJ He YS Jiang J Wang HQ Development of a multiplex MethyLight assay for the detection of multigene methylation in human colorectal cancer Cancer Genet Cytogenet 2010 202 1 10 10.1016/j.cancergencyto.2010.05.018 20804913

[B20] Lind GE Kleivi K Meling GI Teixeira MR Thiis-Evensen E Rognum TO Lothe RA ADAMTS1, CRABP1, and NR3C1 identified as epigenetically deregulated genes in colorectal tumorigenesis Cell Oncol 2006 28 259 272 17167179 10.1155/2006/949506 PMC4618201

[B21] Lind GE Ahlquist T Lothe RA DNA hypermethylation of MAL: a promising diagnostic biomarker for colorectal tumors Gastroenterology 2007 132 1631 1632 10.1053/j.gastro.2007.03.003 17408629

[B22] Lind GE Ahlquist T Kolberg M Berg M Eknaes M Alonso MA Kallioniemi A Meling GI Skotheim RI Rognum TO Thiis-Evensen E Lothe RA Hypermethylated MAL gene - a silent marker of early colon tumorigenesis J Transl Med 2008 6 13 13 18346269 10.1186/1479-5876-6-13 PMC2292685

[B23] Kleivi K Teixeira MR Eknaes M Diep CB Jakobsen KS Hamelin R Lothe RA Genome signatures of colon carcinoma cell lines Cancer Genet Cytogenet 2004 155 119 131 10.1016/j.cancergencyto.2004.03.014 15571797

[B24] Kunkel LM Smith KD Boyer SH Borgaonkar DS Wachtel SS Miller OJ Breg WR Jones HW Rary JM Analysis of human Y-chromosome-specific reiterated DNA in chromosome variants Proc Natl Acad Sci USA 1977 74 1245 1249 10.1073/pnas.74.3.1245 265567 PMC430660

[B25] Meling GI Lothe RA Børresen AL Hauge S Graue C Clausen OP Rognum TO Genetic alterations within the retinoblastoma locus in colorectal carcinomas. Relation to DNA ploidy pattern studied by flow cytometric analysis Br J Cancer 1991 64 475 480 10.1038/bjc.1991.334 1911187 PMC1977625

[B26] Lothe RA Peltomäki P Meling GI Aaltonen LA Nyström-Lahti M Pylkkänen L Heimdal K Andersen TI Møller P Rognum TO Fosså SD Haldorsen T Langmark F Brøgger A de la CHapelle A Børresen AL Genomic instability in colorectal cancer: relationship to clinicopathological variables and family history Cancer Res 1993 53 5849 5852 8261392

[B27] Thorstensen L Lind GE Løvig T Diep CB Meling GI Rognum TO Lothe RA Genetic and Epigenetic Changes of Components Affecting the WNT Pathway in Colorectal Carcinomas Stratified by Microsatellite Instability Neoplasia 2005 7 99 108 10.1593/neo.04448 15802015 PMC1501125

[B28] Thiis-Evensen E Hoff GS Sauar J Langmark F Majak BM Vatn MH Population-based surveillance by colonoscopy: effect on the incidence of colorectal cancer. Telemark Polyp Study I Scand J Gastroenterol 1999 34 414 420 10.1080/003655299750026443 10365903

[B29] Bretthauer M Gondal G Larsen K Carlsen E Eide TJ Grotmol T Skovlund E Tveit KM Vatn MH Hoff G Design, organization and management of a controlled population screening study for detection of colorectal neoplasia: attendance rates in the NORCCAP study (Norwegian Colorectal Cancer Prevention) Scand J Gastroenterol 2002 37 568 573 10.1080/00365520252903125 12059059

[B30] Kent WJ Sugnet CW Furey TS Roskin KM Pringle TH Zahler AM Haussler D The human genome browser at UCSC Genome Res 2002 12 996 1006 12045153 10.1101/gr.229102 PMC186604

[B31] Weisenberger DJ Campan M Long TI Kim M Woods C Fiala E Ehrlich M Laird PW Analysis of repetitive element DNA methylation by MethyLight Nucleic Acids Res 2005 33 6823 6836 10.1093/nar/gki987 16326863 PMC1301596

[B32] Widschwendter M Siegmund KD Muller HM Fiegl H Marth C Muller-Holzner E Jones PA Laird PW Association of breast cancer DNA methylation profiles with hormone receptor status and response to tamoxifen Cancer Res 2004 64 3807 3813 10.1158/0008-5472.CAN-03-3852 15172987

[B33] Melki JR Vincent PC Clark SJ Concurrent DNA hypermethylation of multiple genes in acute myeloid leukemia Cancer Res 1999 59 3730 3740 10446989

[B34] Toyota M Ahuja N Ohe-Toyota M Herman JG Baylin SB Issa JP CpG island methylator phenotype in colorectal cancer Proc Natl Acad Sci USA 1999 96 8681 8686 10.1073/pnas.96.15.8681 10411935 PMC17576

[B35] Costello JF Fruhwald MC Smiraglia DJ Rush LJ Robertson GP Gao X Wright FA Feramisco JD Peltomäki P Lang JC Schuller DE Yu L Bloomfield CD Caligiuri MA Yates A Nishikawa R Huang H Petrelli NJ Zhang X O'Dorisio MS Held WA Cavenee WK Plass C Aberrant CpG-island methylation has non-random and tumour-type-specific patterns Nat Genet 2000 24 132 138 10.1038/72785 10655057

[B36] Suzuki H Gabrielson E Chen W Anbazhagan R van Engeland M Weijenberg MP Herman JG Baylin SB A genomic screen for genes upregulated by demethylation and histone deacetylase inhibition in human colorectal cancer Nat Genet 2002 31 141 149 10.1038/ng892 11992124

[B37] Shames DS Girard L Gao B Sato M Lewis CM Shivapurkar N Jiang A Perou CM Kim YH Pollack JR Fong KM Lam CL Wong M Shyr Y Nanda R Olopade OI Gerald W Euhus DM Shay JW Gazdar AF Minna JD A genome-wide screen for promoter methylation in lung cancer identifies novel methylation markers for multiple malignancies PLoS Med 2006 3 e486 10.1371/journal.pmed.0030486 17194187 PMC1716188

[B38] Lofton-Day C Model F Devos T Tetzner R Distler J Schuster M Song X Lesche R Liebenberg V Ebert M Molnar B Grutzmann R Pilarsky C Sledziewski A DNA methylation biomarkers for blood-based colorectal cancer screening Clin Chem 2008 54 414 423 10.1373/clinchem.2007.095992 18089654

[B39] Zou H Harrington JJ Shire AM Rego RL Wang L Campbell ME Oberg AL Ahlquist DA Highly methylated genes in colorectal neoplasia: implications for screening Cancer Epidemiol Biomarkers Prev 2007 16 2686 2696 10.1158/1055-9965.EPI-07-0518 18086775

[B40] Weisenberger DJ Siegmund KD Campan M Young J Long TI Faasse MA Kang GH Widschwendter M Weener D Buchanan D Koh H Simms L Barker M Leggett B Levine J Kim M French AJ Thibodeau SN Jass J Haile R Laird PW CpG island methylator phenotype underlies sporadic microsatellite instability and is tightly associated with BRAF mutation in colorectal cancer Nat Genet 2006 38 787 793 10.1038/ng1834 16804544

[B41] Chung W Kwabi-Addo B Ittmann M Jelinek J Shen L Yu Y Issa JP Identification of novel tumor markers in prostate, colon and breast cancer by unbiased methylation profiling PLoS ONE 2008 3 e2079 10.1371/journal.pone.0002079 18446232 PMC2323612

[B42] Ausch C Kim YH Tsuchiya KD Dzieciatkowski S Washington MK Paraskeva C Radich J Grady WM Comparative analysis of PCR-based biomarker assay methods for colorectal polyp detection from fecal DNA Clin Chem 2009 55 1559 1563 10.1373/clinchem.2008.122937 19541867

[B43] Kim MS Chang X Nagpal JK Yamashita K Baek JH Dasgupta S Wu G Osada M Woo JH Westra WH Trink B Ratovitski EA Moon C Sidransky D The N-methyl-D-aspartate receptor type 2A is frequently methylated in human colorectal carcinoma and suppresses cell growth Oncogene 2008 27 2045 2054 10.1038/sj.onc.1210842 17922030

[B44] Zitt M Zitt M Muller HM DNA methylation in colorectal cancer--impact on screening and therapy monitoring modalities? Dis Markers 2007 23 51 71 17325426 10.1155/2007/891967 PMC3851076

[B45] Huang Z Li L Wang J Hypermethylation of SFRP2 as a potential marker for stool-based detection of colorectal cancer and precancerous lesions Dig Dis Sci 2007 52 2287 2291 10.1007/s10620-007-9755-y 17410438

[B46] Oberwalder M Zitt M Wöntner C Fiegl H Goebel G Zitt M Köhle O Mühlmann G Öfner D Margreiter R Müller HM SFRP2 methylation in fecal DNA-a marker for colorectal polyps Int J Colorectal Dis 2007 15 9 10.1007/s00384-007-0355-2 17639423

[B47] Wang DR Tang D Hypermethylated SFRP2 gene in fecal DNA is a high potential biomarker for colorectal cancer noninvasive screening World J Gastroenterol 2008 14 524 531 10.3748/wjg.14.524 18203283 PMC2681142

[B48] Devos T Tetzner R Model F Weiss G Schuster M Distler J Steiger KV Grutzmann R Pilarsky C Habermann JK Fleshner PR Oubre BM Day R Sledziewski AZ Lofton-Day C Circulating Methylated SEPT9 DNA in Plasma Is a Biomarker for Colorectal Cancer Clin Chem 2009 10.1373/clinchem.2008.115808 19406918

[B49] Lee B Godfrey M Vitale E Hori H Mattei MG Sarfarazi M Tsipouras P Ramirez F Hollister DW Linkage of Marfan syndrome and a phenotypically related disorder to two different fibrillin genes Nature 1991 352 330 334 10.1038/352330a0 1852206

[B50] Wang Y Yu Q Cho AH Rondeau G Welsh J Adamson E Mercola D McClelland M Survey of differentially methylated promoters in prostate cancer cell lines Neoplasia 2005 7 748 760 10.1593/neo.05289 16207477 PMC1501885

[B51] Hellebrekers DM Melotte V Vire E Langenkamp E Molema G Fuks F Herman JG van Criekinge W Griffioen AW van Engeland M Identification of epigenetically silenced genes in tumor endothelial cells Cancer Res 2007 67 4138 4148 10.1158/0008-5472.CAN-06-3032 17483324

[B52] Jowaed A Schmitt I Kaut O Wullner U Methylation regulates alpha-synuclein expression and is decreased in Parkinson's disease patients' brains J Neurosci 2010 30 6355 6359 10.1523/JNEUROSCI.6119-09.2010 20445061 PMC6632710

[B53] Yan PS Venkataramu C Ibrahim A Liu JC Shen RZ Diaz NM Centeno B Weber F Leu YW Shapiro CL Eng C Yeatman TJ Huang TH Mapping geographic zones of cancer risk with epigenetic biomarkers in normal breast tissue Clin Cancer Res 2006 12 6626 6636 10.1158/1078-0432.CCR-06-0467 17121881

[B54] Lind GE Danielsen SA Ahlquist T Merok MA Rognum TO Meling GI Bretthauer M Thiis-Evensen E Nesbakken A Lothe RA A novel epigenetic biomarker panel for early detection of colorectal cancer and adenomas Ejc Supplements 2009 7 145

[B55] Kilpinen S Autio R Ojala K Iljin K Bucher E Sara H Pisto T Saarela M Skotheim TI Bjorkman M Mpindi JP Haapa-Paananen S Vainio P Edgren H Wolf M Astola J Nees M Hautaniemi S Kallioniemi O Systematic bioinformatic analysis of expression levels of 17,330 human genes across 9,783 samples from 175 types of healthy and pathological tissues Genome Biol 2008 9 R139 10.1186/gb-2008-9-9-r139 18803840 PMC2592717

