# Supplementary material for: Identification of an epigenetic biomarker panel with high sensitivity and specificity for colorectal cancer and adenomas
Source: Mol Cancer. 2011 Jul 21;10:85. doi: 10.1186/1476-4598-10-85 (PMC3166273; doi:10.1186/1476-4598-10-85)
Supplement: Additional file 1 — Supplemental tables. [file 1476-4598-10-85-S1.DOC]

***Additional file 1***

**Cancer cell lines**

In the present study 29 cell lines from various cancer tissues other than colon cancer were included (breast, BT20, BT-474, Hs578, SK-BR-3, T47D, ZR-75-1, ZR-75-30; gastric, AGS, KATO III, NCI-N87; kidney, 786-O, ACHN, Caki-1, Caki-2; ovary, ES-2, OV-90, OVCAR-3, SK-OV-3; pancreas, AsPC-1, BxPC-3, CFPAC-1, HPAFII, PaCa-2, Panc-1; prostate, LNCaP; and uterus, AN3CA, HEC-1-A, KLE, RL95-2). The cell lines were cultured according to the recommendations provided by the American Type Culture Collection (ATCC). We have previously authenticated the colon cancer cell lines by a combined molecular cytogenetic profiling [23]. Cancer cell lines ACHN, AGS, AN3CA, BT20, Caki-1, Caki-2, ES-2, HEC-1-A, Hs578, KATO III, KLE, LNCaP, NCI-N87, OV-90, RL95-2, T47D, and 786-O have been purchased from ATCC in the period between 2005 and 2006 and have been cultured a limited number of passages.

***Additional Tables***

**Table S1.** Primers used for qualitative Methylation-Specific Polymerase Chain Reaction (MSP) and bisulfite sequencing**.** ^a^When *C2orf32* (hg17) was annotated *CNRIP1* (hg18), the transcription start point of NM_015463 was moved 340 bases upstream. The original methylated MSP primer set was located -188 to -14 relative to the transcription start point.

^b^From hg17 to hg18, the transcription start point of NM_06832 (originally named *PLEKHC1*, now named *FERMT2*) has been moved 105 bp upstream. The methylated MSP primers were originally designed to be located -92 to 14 relative to the transcription start point.

^c^For *MEF2C* the initial methylated MSP assay was located from -190 to 82, however these primers resulted in an unspecific PCR product hence a second primer pair was designed within the first exon of the gene.

Abbreviations: MSP, methylation-specific polymerase chain reaction; BS, bisulfite sequencing; M, methylated-specific primers; U, unmethylated-specific primers; Frg. Size, fragment size (in bases); An. Temp, annealing temperature (in degrees celsius). Fragment location lists the start and end point (in bases) of each fragment relative to the transcription start point provided by the USCS Genome Browser [http://genome.ucsc.edu/](http://genome.ucsc.edu/index.html).

| **Primer Set** | **Forward Primer** | **Reverse Primer** | **Frg. Size** | **MgCl mM** | **An. Temp** | **Fragment Location** | **Accession Number** |
| --- | --- | --- | --- | --- | --- | --- | --- |
| BEX1 MSP-M | AGTTAATTGGTCGTCGGTTC | CGAATAACGACTACACCGAA | 121 | 1.5 | 51 | -47 to 74 | NM_018476 |
| BEX1 MSP-U | ATTAGTTAATTGGTTGTTGGTTT | ACACAAATAACAACTACACCAAA | 127 | 1.5 | 51 | -50 to 77 |  |
| BEX1 BS | ATTTGTGGGTTTTTAGATTGGA | CCAAAAAACCACTATATTCCCA | 447 | 1.5 | 53 | -163 to 284 |  |
| C3orf14 MSP-M | GTAATTTAGATTTCGGAGGGC | CGACCAAAAAAAACGAAAA | 124 | 1.5 | 53 | -292 to -168 | NM_020685 |
| C3orf14 MSP-U | TTTGTAATTTAGATTTTGGAGGGT | CCAACCAAAAAAAACAAAAACA | 128 | 1.7 | 53 | -295 to -167 |  |
| CNRIP1 MSP-M | TCGTTTTTTGGTATAGTGGTC | CAAATCCGCGCAACTAAA | 174 | 1.5 | 52 | 152 to 326 | NM_015463^a^ |
| CNRIP1 MSP-U | GTTTTGTTTTTTGGTATAGTGGTT | CAAATCCACACAACTAAAAAC | 177 | 1.5 | 52 | 149 to 326 |  |
| CNRIP1 BS | TTTTAYGTAGTTGGTYGAGG | CTCCTTAAACTATAACCCCCCT | 360 | 1.5 | 48 | 110 to 470 |  |
| COL15A1 MSP-M | TAGAGGCGAGCGTACGTTC | GACGAACAAAATCCCGACTC | 174 | 1.5 | 57 | -111 to 63 | NM_001855 |
| COL15A1 MSP-U | TAGTAGAGGTGAGTGTATGTTT | AACAAACAAAATCCCAACTCCCA | 177 | 1.5 | 57 | -114 to 63 |  |
| FBN1 MSP-M | GTATTTTTTTCGCGAGAAATC | AATCGTAACCGCTACAACC | 164 | 2.0 | 48 | -66 to 98 | NM_000138 |
| FBN1 MSP-U | AAAGTATTTTTTTTGTGAGAAATT | CCCAATCATAACCACTACAACC | 170 | 1.7 | 48 | -69 to 101 |  |
| FBN1 BS | TTTTTTTTTTTTAAAAAAAGTATTTTT | CCRAACCAAAAAACTATCA | 410 | 3.0 | 48 | -85 to 325 |  |
| FERMT2 MSP-M | GGAGTTAGGCGAGGGGTC | ACTCGCGAAACGACGATAT | 106 | 1.5 | 59 | 13 to 119 | NM_006832^b^ |
| FERMT2 MSP-U | GGGGAGTTAGGTGAGGGGTT | ACTCACAAAACAACAATATCC | 108 | 1.5 | 53 | 11 to 119 |  |
| FHL1 MSP-M | TCGTGTAGTGGGTAGAGTTC | CTCCGCCGAACGATAAAT | 165 | 1.5 | 55 | -160 to 5 | NM_001449 |
| FHL1 MSP-U | TTTTTGTGTAGTGGGTAGAGTTT | CCCCTCCACCAAACAATAAAT | 171 | 1.5 | 55 | -163 to 8 |  |
| INA MSP-M | AGGAGTTTCGTTTTTAGCGC | ACGACTTCAACGCGAACTAC | 118 | 1.5 | 55 | -93 to 25 | NM_032727 |
| INA MSP-U | AGTAGGAGTTTTGTTTTTAGTGT | ACAACTTCAACACAAACTACAAA | 121 | 1.5 | 55 | -96 to 25 |  |
| INA BS | TYGGTTAGATTTTGAGTAGGAG | AATACTCCRAACCRAAACTC | 181 | 1.5 | 52 | -110 to 71 |  |
| KCNQ2 MSP-M | AGGTGGTCGTAGCGTTTTC | CCCGAATCGAACTCAAACT | 149 | 1.5 | 55 | 9 to 158 | NM_172106 |
| KCNQ2 MSP-U | GAGGTGGTTGTAGTGTTTTT | CCCCAAATCAAACTCAAACT | 151 | 1.5 | 55 | 8 to 159 |  |
| LEF1 MSP-M | TTTATTGCGGAGTTTTCGC | GCTTCGATTTTTCTTCTCGAA | 144 | 2.0 | 51 | -162 to -18 | NM_016269 |
| LEF1 MSP-U | TTTTTTATTGTGGAGTTTTTGT | ACTTCAATTTTTCTTCTCAAAACT | 147 | 2.0 | 51 | -165 to -18 |  |
| MEF2C MSP-M | AGTTTTTTGTTCGTTTTGTTC | TTTACTTCGTCCAACGTTAA | 162 | 1.7 | 50 | 2 to 164 | NM_002397^c^ |
| MEF2C MSP-U | TTTAGTTTTTTGTTTGTTTTGTTT | CTTTACTTCATCCAACATTAAAA | 166 | 1.7 | 50 | -1 to 165 |  |
| SNCA MSP-M | CGGGTTGTAGCGTAGATTTC | CGTCGAATAACCACTCCC | 125 | 1.5 | 53 | -108 to 17 | NM_000345 |
| SNCA MSP-U | GTGTGGGTTGTAGTGTAGATTTT | TCATCAAATAACCACTCCCAA | 129 | 1.5 | 53 | -111 to 18 |  |
| SNCA BS | AGAAGGGGTTTAAGAGAGG | ACTATCCCCAAAAAAAACC | 260 | 1.5 | 53 | -169 to 91 |  |
| UBE3A MSP-M | CGTTGTTTGTCGGGATATTC | CCCGTCGTCTCCTATAATCA | 122 | 1.5 | 56 | -76 to 46 | NM_130839 |
| UBE3A MSP-U | GTGTTGTTTGTTGGGATATTT | CCCCATCATCTCCTATAATCA | 124 | 1.5 | 56 | -77 to 47 |  |

**Table S2.** Primers used for quantitative Methylation-Specific Polymerase Chain Reaction (qMSP).

| **Assay** | **Forward Primer** | **Reverse Primer** | **Probe** | **Frg. Size** |
| --- | --- | --- | --- | --- |
| ALU qMSP | GGTTAGGTATAGTGGTTTATATTTGTAATTTTAGTA | ATTAACTAAACTAATCTTAAACTCCTAACCTCA | 6FAM-CCTACCTTAACCTCCC-MGB | 98 |
| CNRIP1 qMSP | TTTAGTTGCGCGGATTTGC | GCACCCGAAAACTCGCTCTA | 6FAM-CCGCAAACCGCCG-MGB | 55 |
| FBN1 qMSP | GAGTTATAGTTGGGATAGTTGCGAGC | AACGACGACTCCGACTCCC | 6FAM-CGCTACAACCACTACTCGA-MGB | 101 |
| INA qMSP | AAAAGTCGGGCGTATCGTTTC | CGACTTCAACGCGAACTACAAA | 6FAM-ATACGACAAACAAAACGCGA-MGB | 75 |
| MAL qMSP | CGTTTAGGTTATTGGGTTTCGC | CGAACGCCGCTCAAACTC | 6FAM-CGCAAACCTCTCGCTAA-MGB | 63 |
| SNCA qMSP | GCGTTTTGGGCGTTTTTTTAC | CGCTATAAACCGACGACGC | 6FAM-CGCTAACCTATCGTCGAA-MGB | 143 |
| SPG20 qMSP | GCGCGTCGTGGAACGT | CTACGCTCGCCGAAAACC | 6FAM-CGCGCTTACCGTAACAA-MGB | 84 |

Abbreviations: Frg. Size, fragment size in bases; qMSP, quantitative methylation-specific polymerase chain reaction; MGB, minor groove binder. See Figure 6 for primer and probe locations. Primers and probe for the *SPG20* qMSP assay have been described previously [9].

**Table S3.** Receiver Operating Characteristics (ROC) curve analysis.

| **Samples** | **Biomarkers** | **AUC** | **Std. error** | **Asymptotic sig.** | **95%C.I.lower** | **95%C.I.upper** |
| --- | --- | --- | --- | --- | --- | --- |
|  | *CNRIP1* | 0.993 | 0.007 | 9.3E-21 | 0.979 | 1.007 |
| CRC Test Set | *FBN1* | 0.950 | 0.019 | 1.3E-17 | 0.913 | 0.987 |
|  | *INA* | 0.897 | 0.029 | 5.3E-14 | 0.840 | 0.954 |
|  | *MAL* | 0.980 | 0.012 | 8.4E-20 | 0.957 | 1.004 |
|  | *SNCA* | 0.876 | 0.032 | 1.0E-12 | 0.814 | 0.938 |
|  | Panel | 0.999 | 0.001 | 3.1E-21 | 0.996 | 1.001 |
|  | *CNRIP1* | 0.957 | 0.016 | 2.9E-22 | 0.926 | 0.989 |
| CRC Validation Set | *FBN1* | 0.884 | 0.028 | 3.5E-16 | 0.829 | 0.940 |
|  | *INA* | 0.851 | 0.029 | 9.3E-14 | 0.793 | 0.909 |
|  | *MAL* | 0.944 | 0.019 | 4.0E-21 | 0.906 | 0.982 |
|  | *SNCA* | 0.687 | 0.043 | 7.4E-05 | 0.602 | 0.773 |
|  | Panel | 0.963 | 0.016 | 8.8E-23 | 0.932 | 0.994 |
|  | ***CNRIP1*** | **0.976** | **0.009** | **4.1E-42** | **0.959** | **0.993** |
| **CRCs Combined (Test and Validation Sets)** | ***FBN1*** | **0.914** | **0.017** | **3.0E-32** | **0.880** | **0.948** |
|  | ***INA*** | **0.873** | **0.021** | **2.0E-26** | **0.832** | **0.913** |
|  | ***MAL*** | **0.964** | **0.011** | **5.5E-40** | **0.942** | **0.985** |
|  | ***SNCA*** | **0.776** | **0.028** | **3.0E-15** | **0.722** | **0.830** |
|  | **Panel** | **0.984** | **0.007** | **1.9E-43** | **0.971** | **0.998** |
|  | *CNRIP1* | 0.976 | 0.012 | 7.0E-18 | 0.953 | 0.999 |
| Adenomas Test Set | *FBN1* | 0.949 | 0.020 | 4.5E-16 | 0.909 | 0.989 |
|  | *INA* | 0.925 | 0.026 | 1.5E-14 | 0.873 | 0.976 |
|  | *MAL* | 0.967 | 0.017 | 2.9E-17 | 0.934 | 1.000 |
|  | *SNCA* | 0.820 | 0.041 | 6.7E-09 | 0.740 | 0.900 |
|  | Panel | 0.981 | 0.011 | 3.2E-18 | 0.960 | 1.002 |
|  | *CNRIP1* | 0.947 | 0.027 | 7.7E-16 | 0.894 | 1.000 |
| Adenomas Validation Set | *FBN1* | 0.903 | 0.032 | 3.7E-13 | 0.840 | 0.966 |
|  | *INA* | 0.704 | 0.051 | 2.3E-04 | 0.604 | 0.804 |
|  | *MAL* | 0.912 | 0.034 | 1.0E-13 | 0.847 | 0.978 |
|  | *SNCA* | 0.594 | 0.064 | 8.9E-02 | 0.470 | 0.719 |
|  | Panel | 0.962 | 0.025 | 8.2E-17 | 0.913 | 1.010 |
|  | ***CNRIP1*** | **0.954** | **0.015** | **1.8E-31** | **0.924** | **0.984** |
| **Adenomas Combined (Test and Validation Sets)** | ***FBN1*** | **0.922** | **0.018** | **2.2E-27** | **0.886** | **0.958** |
|  | ***INA*** | **0.825** | **0.028** | **7.1E-17** | **0.769** | **0.881** |
|  | ***MAL*** | **0.945** | **0.017** | **3.0E-30** | **0.912** | **0.978** |
|  | ***SNCA*** | **0.714** | **0.037** | **3.6E-08** | **0.642** | **0.786** |
|  | **Panel** | **0.968** | **0.013** | **2.6E-33** | **0.943** | **0.993** |

ROC curves for individual biomarkers were generated using percentage methylated reference (PMR) values and tissue type (carcinoma or adenoma and normal) as input. For the combined biomarker panel (“Panel”) the sum of PMR values for *CNRIP1*, *FBN1*, *INA*, *MAL*, *SNCA*, and *SPG20* was used. Individual ROC values for *SPG20* have been published elsewhere [9]. Abbreviations: Asymptotic sig., asymptotic significance; AUC, area under the curve; C.I, confidence interval; CRC, colorectal carcinoma; Std. error, standard error.

**Table S4. Real-Time PCR analysis of methylated and unmethylated tissue samples.**

| **Gene** | **Methylation Status** | **mRNA Expression (Mean)** | **Std. Deviation** | ***P*- value** |
| --- | --- | --- | --- | --- |
| ***CNRIP1*** | Methylated | 0.395 | 0.219 | **0.001** |
|  | Unmethylated | 1.205 | 1.188 |  |
| ***FBN1*** | Methylated | 0.406 | 0.300 | 0.125 |
|  | Unmethylated | 0.765 | 1.068 |  |
| ***INA*** | Methylated | 0.008 | 0.009 | **0.032** |
|  | Unmethylated | 0.463 | 0.080 |  |
| ***SNCA*** | Methylated | 0.037 | 0.023 | **0.010** |
|  | Unmethylated | 0.166 | 0.220 |  |

mRNA expression has been normalized to the expression of *GUSB* and *ACTB*. All samples have been analyzed in triplicates and the median value used for analysis. Student T-test has been used to calculate *P*-values. An association between reduced gene expression and promoter methylation of *MAL* [22] and *SPG20* [9] has been described previously.
