# Supplementary figures and images for: Identification of an epigenetic biomarker panel with high sensitivity and specificity for colorectal cancer and adenomas
Source: Mol Cancer. 2011 Jul 21;10:85. doi: 10.1186/1476-4598-10-85 (PMC3166273; doi:10.1186/1476-4598-10-85)

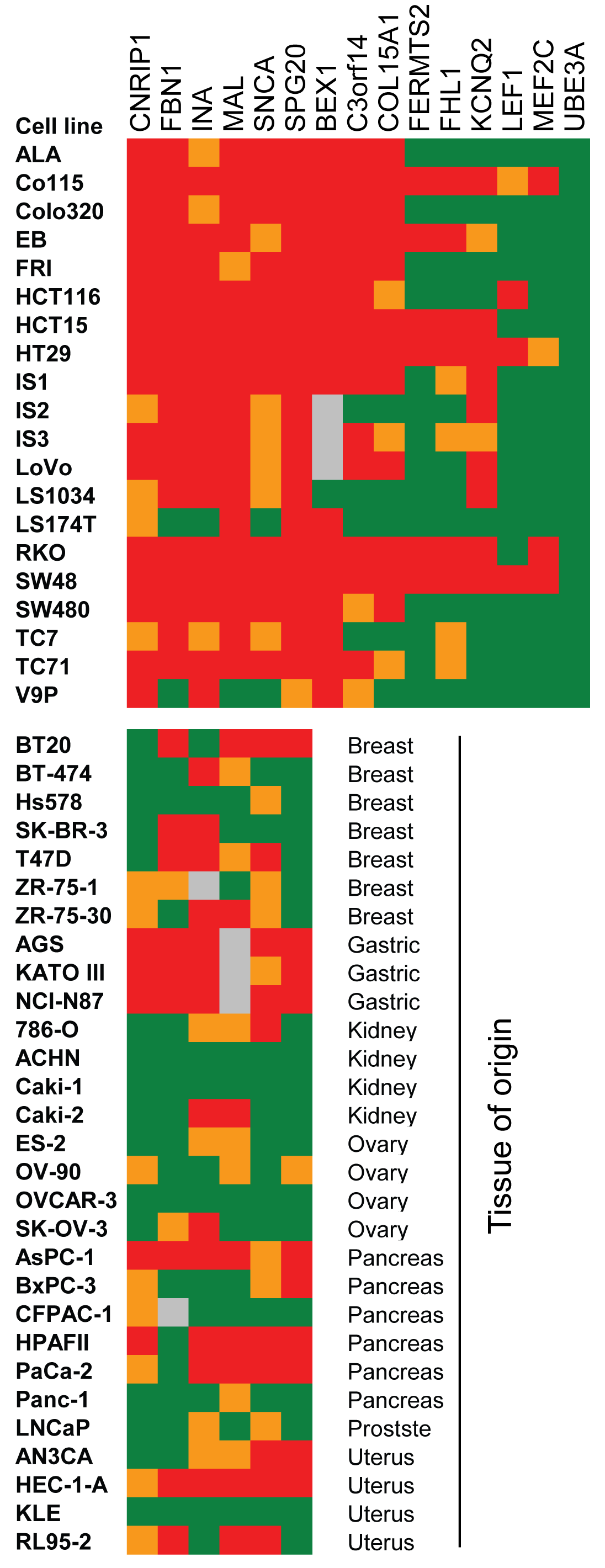

Supplement: Additional file 2 — Figure S1. Summary of methylation-specific polymerase chain reaction (MSP) results in cancer cell lines. Red color indicates methylated sample, orange color indicates the presence of both methylated and unmethylated bands, green color indicates unmethylated sample, and grey color indicates samples that were not successfully amplified/analyzed. The upper part of the figure illustrates the results from colon cancer cell lines (n = 20) and the lower part of the figure from cell lines originating from various cancer tissues (n = 29). [file 1476-4598-10-85-S2.TIFF]
